# Analysis of mRNA‑lncRNA and mRNA‑lncRNA-pathway co‑expression networks based on WGCNA in developing pediatric sepsis

**DOI:** 10.1080/21655979.2021.1908029

**Published:** 2021-05-05

**Authors:** Xiaojuan Zhang, Yuqing Cui, Xianfei Ding, Shaohua Liu, Bing Han, Xiaoguang Duan, Haibo Zhang, Tongwen Sun

**Affiliations:** a General ICU, Zhengzhou Key Laboratory of Sepsis, Henan Engineering Research Center for Critical Care Medicine, the First Affiliated Hospital of Zhengzhou University, Henan Key Laboratory of Critical Care Medicine, Zhengzhou, China; b Interdepartmental Division of Critical Care Medicine, Departments of Anesthesia and Physiology, University of Toronto, Toronto, Canada

**Keywords:** Pediatric sepsis, mRNA, lncRNA, WGCNA; diagnostic markers

## Abstract

Pediatric sepsis is a great threat to death worldwide. However, the pathogenesis has not been clearly understood until now in sepsis. This study identified differentially expressed mRNAs and lncRNAs based on Gene Expression Omnibus (GEO) database. And the weighted gene co-expression network analysis (WGCNA) was performed to explore co-expression modules associated with pediatric sepsis. Then, Gene Ontology (GO), KEGG (Kyoto Encyclopedia of Genes and Genomes) pathway, mRNA‑lncRNA and mRNA‑lncRNA-pathway co-expression network analysis was conducted in selected significant module. A total of 1941 mRNAs and 225 lncRNAs were used to conduct WGCNA. And turquoise module was selected as a significant module that was associated with particular traits. The mRNAs functions associated with many vital processes were also shown by GO and KEGG pathway analysis in the turquoise module. Finally, 15 mRNAs (MAPK14, ITGAM, HK3, ALOX5, CR1, HCK, NCF4, PYGL, FLOT1, CARD6, NLRC4, SH3GLB1, PGS1, RAB31, LTB4R) and 4 lncRNAs (GSEC, NONHSAT160878.1, XR_926068.1 and RARA-AS1) were selected as hub genes in mRNA‑lncRNA-Pathway co-expression network. We identified 15 mRNAs and 4 lncRNAs as diagnostic markers, which have potential functions in pediatric sepsis. Our study provides more directions to study the molecular mechanism of pediatric sepsis.

**Abbreviations**: mRNA: messenger RNA; lncRNA: long noncoding RNAs; GEO: Gene Expression Omnibus; WGCNA: weighted gene co-expression network analysis; GO: Gene Ontology; KEGG: Kyoto Encyclopedia of Genes and Genomes; SIRS: systemic inflammatory response syndrome; TOM: topological overlap measure; BP: biological process; MF: molecular function; CC: cellular component; ROC: receiver operating characteristic curve; AUC: area under curve; MAPK14: Mitogen-activated protein kinase 14; ALI: acute lung injury; ITGAM: Integrin subunit alpha M; HK3: Hexokinase 3; LPS: lipopolysaccharide; 5-LO: 5-lipoxygenase; LTs: leukotrienes; LTB4R: leukotriene B4 receptor.

## Graphical abstract

**Figure:**
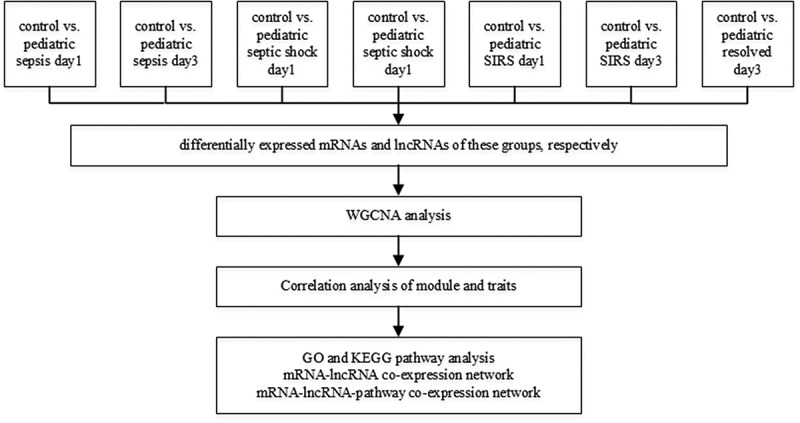

## Introduction

There are about 1.2 million childhood sepsis cases to be diagnosed globally per year [1]. Unfortunately, mortality was reported to be as high as 25%-50% for children hospitalized for sepsis [2]. The definition of sepsis has been revised repeatedly, and the latest adult sepsis definition cannot be rigidly applied to children, so the early diagnosis and appropriate managements essential to improve clinical outcomes for children at risk for sepsis [3]. Therefore, understanding the potential molecular mechanism underlying sepsis is critical to identify early diagnostic biomarkers and find effective drugs.

Long noncoding RNAs (lncRNAs) are a type of non-protein transcripts that exceed 200 nucleotides in length [4]. LncRNAs may be involved in messenger RNAs (mRNAs) splicing and maturation, mRNAs transport or localization, mRNAs stabilization [5]. There was increasing evidences that lncRNAs had critical regulatory effects on the pathophysiology and organ dysfunction in sepsis [6]. For instance, lncRNA NEAT1 played a vital role in inducing acute kidney injury by modulating NF-ΚB pathway. LncRNA ENST00000504301.1 and ENST00000452391.1 were reported had differential expression between sepsis survivors and non-survivors [7,8]. Besides, there has been a number of recent work that use machine learning methods to identify novel genes in pediatric sepsis. Mohammed et al identified 53 differentially expressed mRNAs from 181 septic shock patients [9]. And Manatakis et al introduced Transcriptomic Signature Distance (TSD) and evaluated its performance using publicly available RNA-seq data sets [10]. Banerjee et al conducted a meta-analysis of published gene expression datasets to train an artificial intelligence system and found eight early biomarkers to predict the severity of sepsis or death [11]. But these reports only showed the effects of mRNA and lncRNA alone in sepsis.

To explore the interaction mRNA-lncRNA and diagnostic markers in pediatric sepsis, we first identified mRNAs and lncRNAs based on RNA-seq datasets of the whole spectrum of systemic inflammatory response syndrome (SIRS), sepsis, septic shock, SIRS resolved and control group at day 1 and day 3 from Gene Expression Omnibus (GEO) database. Then, weighted gene co-expression network analysis (WGCNA) and module–trait relationships were conducted to select important modules. Subsequently, function and pathway enrichment analyses of module were conducted by Gene Ontology (GO) and Kyoto Encyclopedia of Genes and Genomes (KEGG). mRNA‑lncRNA and mRNA‑lncRNA-Pathway networks also were constructed in significant modules. No study performed WGCNA to systematically construct mRNA‑lncRNA-Pathway co-expression networks concerning pediatric sepsis, which may provide a better understanding for lncRNA biological function and to find related biomarkers.

## Methods

### Data Collection

Expression dataset of sepsis (GSE13904) were downloaded from the GEO database (https://www.ncbi.nlm.nih.gov/geo/query/acc.cgi?acc=GSE13904) and then collated into standardized raw data for subsequent analysis. The mRNAs and lncRNAs expression data of 227 children included 18 control samples, 32 sepsis samples, 67 septic shock samples, and 22 SIRS samples on day 1. The day 3 samples consisted of 20 sepsis patients, 39 septic shock patients, 24 patients meeting SIRS resolved and patients had paired day 1 and day 3.

mRNA and lncRNA screening

Limma package in R was used to find mRNA and lncRNA between the four related symptoms (sepsis, septic shock, SIRS and SIRS resolved) and control samples at two different time points (day 1, day 3). The differential screening parameter was set as: *P* < 0.05 & false discovery rate (FDR) <0.05 & fold change (FC) >2 to obtain the mRNA and lncRNA for WGCNA analysis.

### Construction of WGCNA network

WGCNA is a method that summarized gene expression data into co-expression modules. It has an obvious advantage to find the complex relationships between relating modules and associated with traits [12]. The 1941 mRNAs and 225 lncRNAs were used to construct a weighted correlation network by WGCNA package of R software. To calculate the link strength between nodes i and j from co-expression network, the Pearson’s correlation coefficient cor (i, j) was defined. Then, the weighted adjacency matrix a_ij_ was calculated as follows: a_ij_ = (0.5 × (1 + cor (i, j)))^β^. β was a soft threshold power, which mostly be concerned with the independence and the average connectivity degree in co-expression modules. The topological overlap measure (TOM) represented the overlap of network neighbors, and (1-TOM) retrieved a pairwise distance to identify hierarchical clustering nodes and modules [13]. Clustering graphs were performed by application of dynamic tree cutting technique of pheatmap package in R software.

### Module–trait relationship analysis

The correlation between co-expression modules and clinical traits was estimated based on the phenotypic information of pediatric sepsis, septic shock, SIRS, SIRS resolved at day 1 and day 3 and control group. A significant co-expression module highly related to traits was identified. Module–trait relationships were computed by Pearson’s correlation tests, and *P* < 0.05 was defined significant correlation.

### Functional enrichment analysis of significant co-expression modules

DAVID (http://david.abcc.ncifcrf.gov/) was a database for annotation, visualization, and integrated discovery. GO and KEGG pathway analysis of mRNAs were conducted by DAVID. The ontology contained three classes: biological process (BP), molecular function (MF), and cellular component (CC). Fisher exact test, and multiple comparison test were used to calculate the significance level (*p*-value), and *p*-value was corrected by FDR. Thus, the function and pathway of mRNAs is identiﬁed in the significant module.

### Construction of mRNA‑lncRNA network and mRNA‑lncRNA-Pathway

The mRNA‑lncRNA co-expression networks of significant module were carried out to explore the association between mRNA and lncRNA. According to mRNA‑lncRNA network and the significant pathways involved in the regulation of mRNA, the network of mRNA‑lncRNA-Pathway was constructed. The purpose of this chat is to reveal the pathways involved in the regulation of lncRNA, so as to predict possible mechanisms of lncRNA in diseases. Significant correlation pairs were applied to build the network in accordance with Pearson correlation coefficients. And the differential co-expression network chats were visualized and analyzed using Cytoscape software (Version 3.5.1).

### Statistical analysis

Statistical analysis was conducted using R software 3.4.0 and SPSS 19.0. Fisher exact and multiple comparison tests were used to find differences between traits. The receiver operating characteristic (ROC) curve was conducted (glmnet package in R software) to estimate the specificity and sensitivity of potential biomarkers. *p* < 0.05 was supposed to be significant.

## Results

1941 mRNAs and 225 lncRNAs were identified among the four related symptoms (sepsis, septic shock, SIRS, SIRS resolved) and control group. Then they were performed to build the co-expression modules using WGCNA analysis. And turquoise module was selected as a significant module correlated with pediatric sepsis. mRNA‑lncRNA co-expression networks also was conducted, and we found 15 mRNAs (MAPK14, ITGAM, HK3, ALOX5, CR1, HCK, NCF4, PYGL, FLOT1, CARD6, NLRC4, SH3GLB1, PGS1, RAB31, LTB4R) and four lncRNAs (GSEC, NONHSAT160878.1, XR_926068.1, RARA-AS1), which were regarded as hub genes. Therefore, the four lncRNAs were speculated to have common functions on complement and coagulation cascades, phagosome, leukocyte trans-endothelial migration, metabolic pathways, and Insulin signaling pathways in pediatric sepsis. The area under the curve of ROC was above 0.88, indicating that 15 mRNAs and four lncRNAs are likely to have good diagnostic value for pediatric sepsis.

Identification of mRNAs and lncRNAs

Our analysis identified 1110 mRNAs and 111 lncRNAs in the comparison groups control vs. pediatric sepsis day 1, 435 mRNAs and 37 lncRNAs in the groups control vs pediatric sepsis day 3, 1278 mRNAs and 134 lncRNAs in the groups control vs pediatric septic shock day1, 1565 mRNAs and 172 lncRNAs in the groups control vs pediatric septic shock day 3, 760 mRNAs and 77 lncRNAs in the groups control vs pediatric SIRS day 1, 824 mRNAs and 112 lncRNAs in the groups control vs pediatric SIRS day 3, 613 mRNAs and 56 lncRNAs in the groups control vs. pediatric SIRS resolved day 3. Totally, we obtained 1941 mRNAs including 927 (47.8%) up-regulated and 1014 (52.2%) down-regulated mRNAs, and 225 lncRNAs containing 132 (58.7%) up-regulated and 93 (41.3%) down-regulated lncRNAs between the four related symptoms (sepsis, septic shock, SIRS, SIRS resolved) and control group. And these mRNAs and lncRNAs were performed by the hierarchical cluster analysis (Supplemental Figures 1 and 2(a-g).

### Construction of WGCNA network

**Figure 1.**
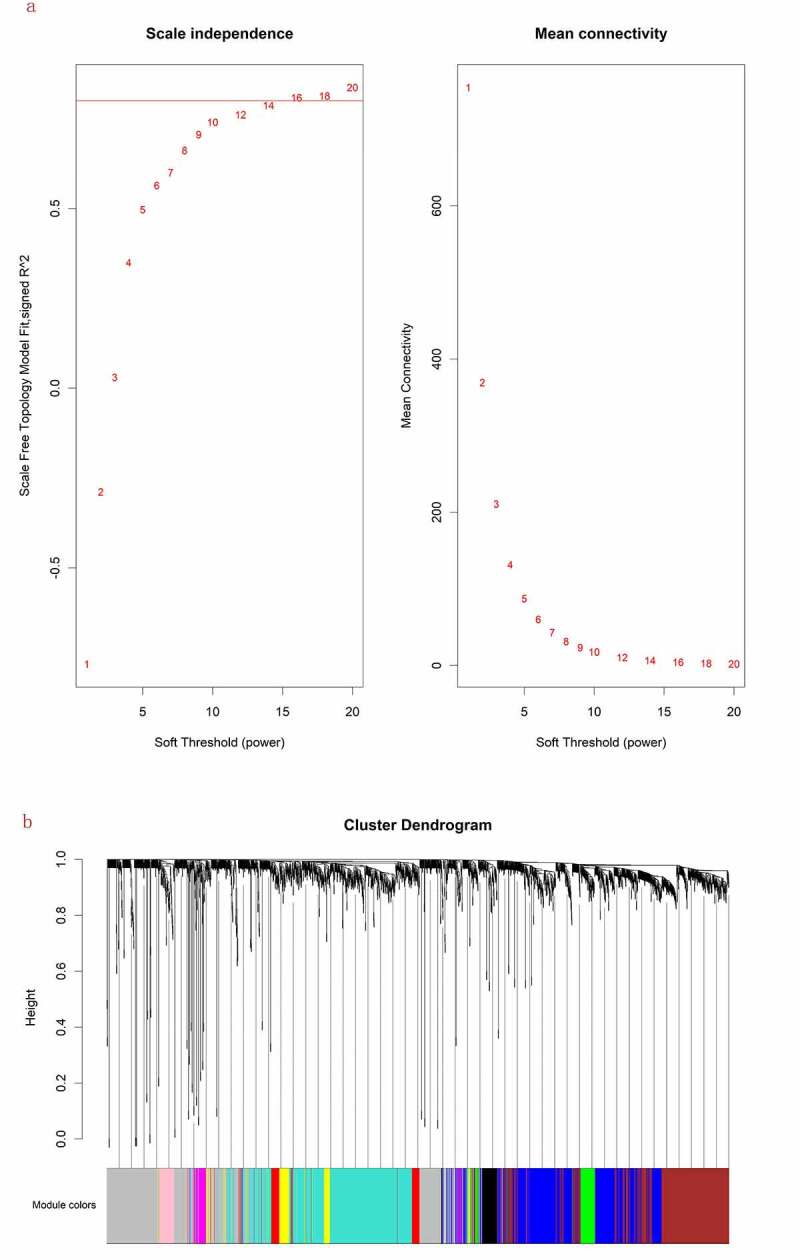
(a) Scale independence and mean connectivity analysis for various soft threshold powers. (b) Clustering dendrograms of mRNAs. Different colors below indicate different co-expression modules

1941 mRNAs and 225 lncRNAs were performed to build the co-expression modules using WGCNA. The cluster analysis was shown that all the samples were in the clusters, so it is not necessary to remove the outlier. The soft threshold power β was set at 16, which was more consistent with scale-free networks and had a more biological significance [14]. The independence had a high degree and the average connectivity had lower level when the power value was equal to 16 (Figure 1a). Therefore, β = 16 was used to engender a hierarchical clustering tree. These mRNAs and lncRNAs with similar expression patterns were put into modules through average link clustering (Figure 1b). Eleven modules (turquoise, gray, blue, brown, yellow, green, red, black, pink, magenta and purple) were recognized and exhibited with different colors totally, and the number of mRNAs and lncRNAs in these modules is shown in (Table 1).

**Table 1.** The number of mRNAs and lncRNAs in the 11 modules

Module	All numbers	mRNAs	lncRNAs
turquoise	494	468	26
gray	482	410	72
blue	410	370	40
brown	380	352	28
yellow	81	71	10
green	72	70	2
red	72	71	1
black	53	49	4
pink	52	50	2
magenta	48	8	40
purple	22		22

### Correlation analysis of module and traits

**Figure 2.**
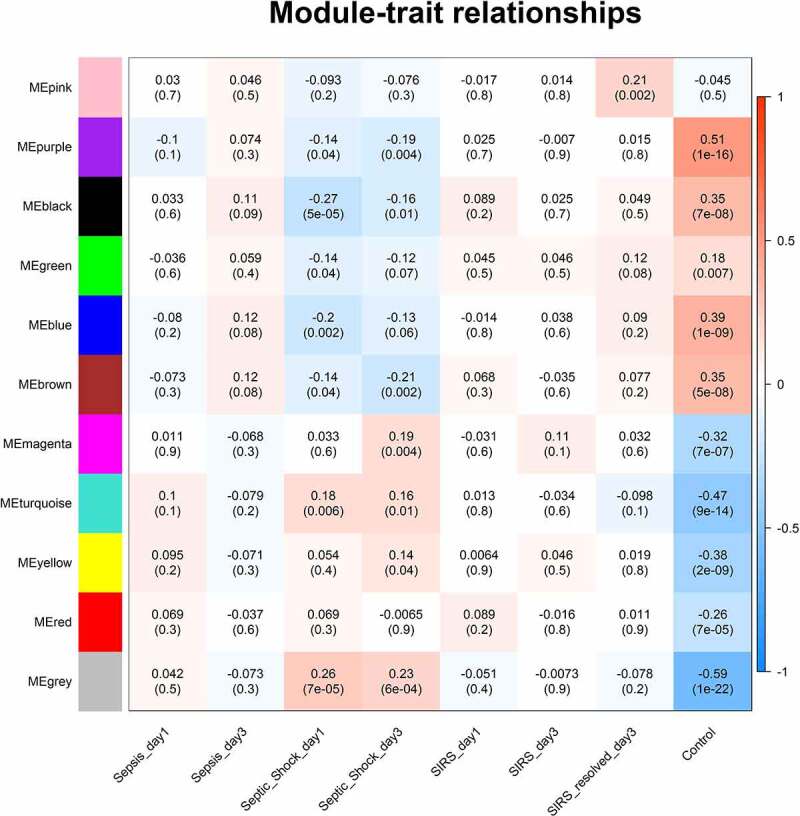
Module–trait relationship. Each row represents a module eigengene and each column represents a trait. Each cell includes the corresponding correlation and *p* value

The relationship between co-expression modules and clinical traits is identiﬁed in Figure 2. We found that the gene change trend of each module was the most consistent between control group and SIRS resolved group, while showed the opposite change between septic shock group and control group. Gray module included genes that did not belong to any module, and the turquoise module was selected as a significant module correlated with pediatric sepsis according to the maximum number of mRNAs and lncRNAs with the most significant *p* value. There were 468 mRNAs and 26 lncRNAs in the turquoise module.

### GO and KEGG pathway analysis

**Figure 3.**
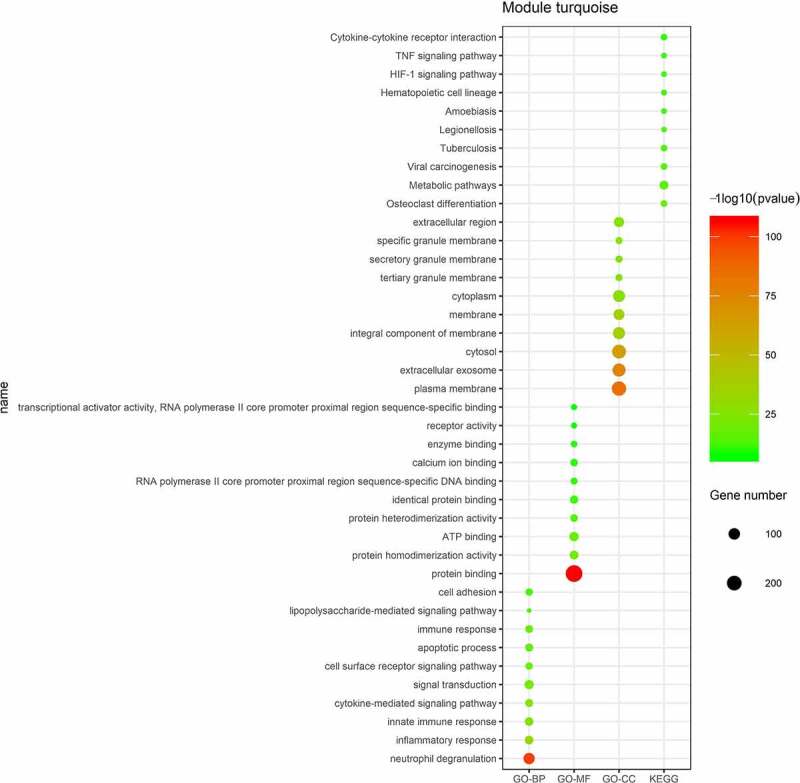
Enriched GO and KEGG pathway analysis of mRNAs in the turquoise module. The size of spots corresponds to the numbers of mRNAs, the color of spots represents *p* value

The mRNAs in the turquoise module were conducted by GO analysis and KEGG pathway enrichment analysis. The results showed that neutrophil degranulation, inflammatory response, innate immune response, and cytokine-mediated signaling pathway were enriched in BP, protein binding, protein homodimerization activity, and ATP binding were mostly involved in MF, plasma membrane, extracellular exosome, and cytosol were enriched in CC. KEGG pathway enrichment analysis suggested that metabolic pathway, TNF signaling pathway and cytokine-cytokine receptor interaction were the most significant pathways (Figure 3).

### Construction of the mRNA‑lncRNA co-expression network

**Figure 4.**
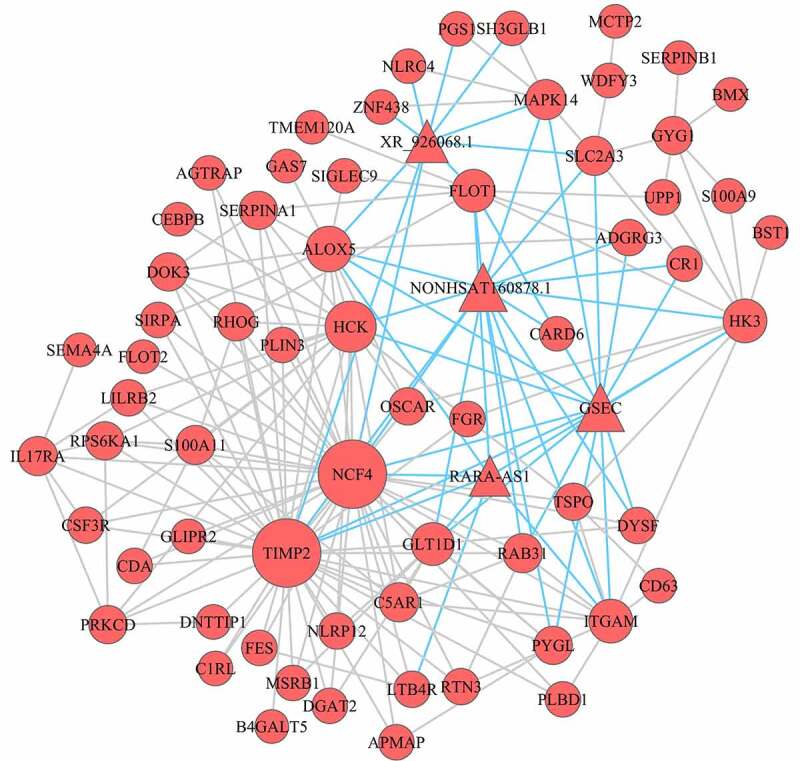
Co-expression mRNA-lncRNA network in the turquoise module. the circular nodes represent the mRNAs, triangle nodes represent lncRNAs. Blue edges represent mRNA-lncRNA interaction and gray edges represent mRNAs–mRNAs interaction

mRNA‑lncRNA co-expression networks were built to understand the roles and functional mechanisms of lncRNA in turquoise module (Figure 4). We found that 64 mRNAs and 4 lncRNAs were inter-regulated and one lncRNA was co-expressed with multiple mRNAs. Besides, multiple lncRNAs also had co-expressed with one mRNA, which suggested that a complicated regulatory association between mRNAs and lncRNAs existed in differential co-expression network.

### Construction of the mRNA‑lncRNA-Pathway co‑expression network

**Figure 5.**
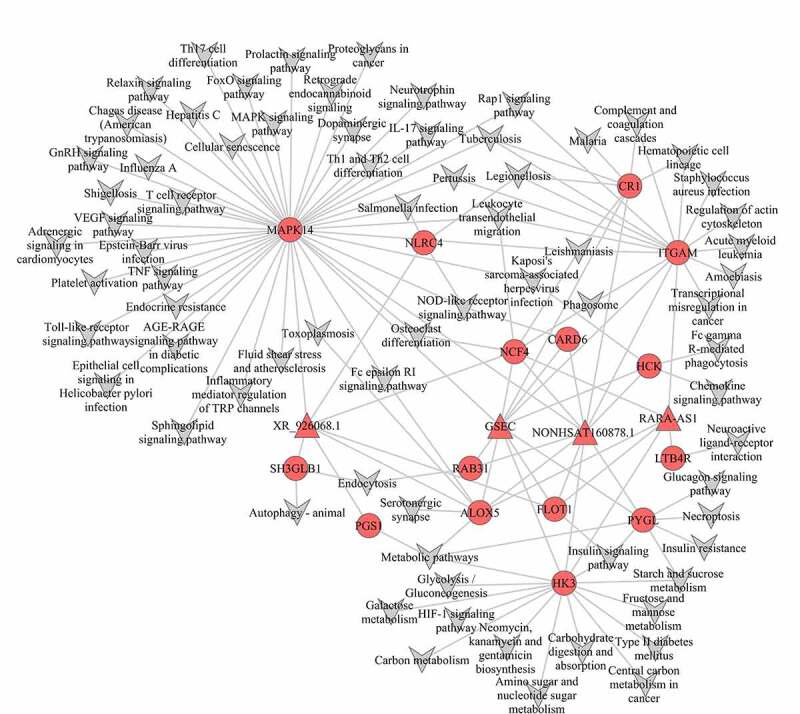
Co-expression mRNA-lncRNA-pathway of the turquoise module. The circular nodes represent the mRNAs, triangle nodes represent lncRNAs

According to the interaction network of mRNA‑lncRNA and mRNA-Pathway, we obtained mRNA‑lncRNA-Pathway networks (Figure 5). In our study, we identified 15 mRNAs and 4 lncRNAs in the mRNA‑lncRNA-Pathway network, and all these genes were up-regulated. In turquoise module, GSEC, NONHSAT160878.1 (two lncRNAs) were linked to eleven mRNAs (MAPK14, ITGAM, HK3, ALOX5, CR1, HCK, NCF4, PYGL, FLOT1, CARD6, RAB31) and enriched in MAPK-signaling pathway, IL-17 signaling pathway, leukocyte trans-endothelial migration, cellular senescence, VEGF signaling pathway, TNF signaling pathway, platelet activation, endocrine resistance, toll-like receptor signaling pathway, inflammatory mediator regulation of TRP channels, complement and coagulation cascades, phagosome, metabolic pathways, Insulin signaling pathway, chemokine signaling pathway, Glucagon signaling pathway, Insulin resistance, NOD-like receptor signaling pathway.

XR_926068.1 (a lncRNA) was related to seven mRNAs (MAPK14, ALOX5, NCF4, FLOT1, NLRC4, SH3GLB1, PGS1) and abundant in MAPK signaling pathway, IL-17 signaling pathway, leukocyte trans-endothelial migration, cellular senescence, VEGF signaling pathway, TNF signaling pathway, platelet activation, endocrine resistance, toll-like receptor signaling pathway, inflammatory mediator regulation of TRP channels, metabolic pathways, phagosome, insulin signaling pathway, metabolic pathways, NOD-like receptor signaling pathway, and autophagy.

RARA-AS1 (a lncRNA) was connected with six mRNAs (ITGAM, HK3, ALOX5, NCF4, FLOT1, LTB4R) enriched in Complement and coagulation cascades, Phagosome, leukocyte trans-endothelial migration, metabolic pathways, and insulin signaling pathways. These four lncRNAs have common function on complement and coagulation cascades, phagosome, leukocyte trans-endothelial migration, metabolic pathways, and insulin signaling pathways in pediatric sepsis.

### Verifying hub mRNA and lncRNA of diagnostic value

**Figure 6.**
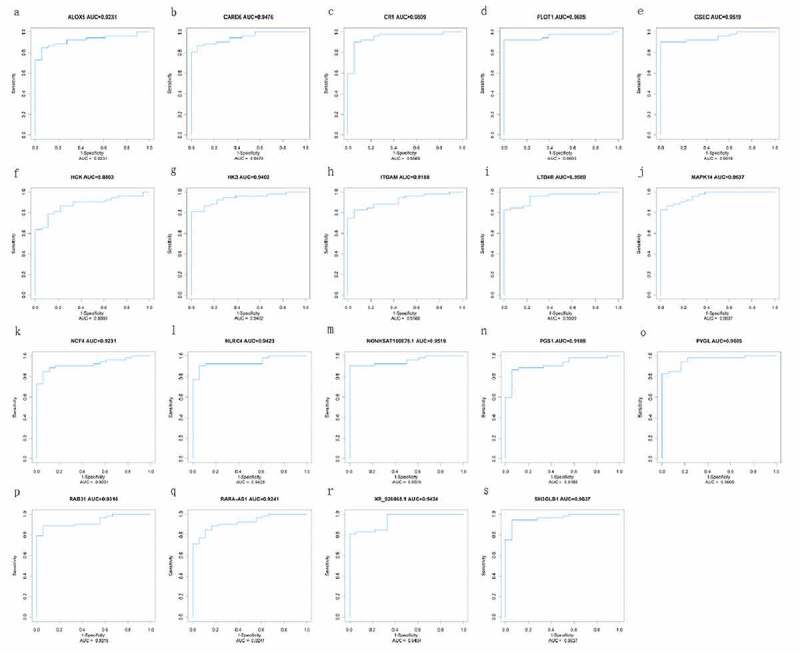
ROC curve of diagnosis of pediatric sepsis. the AUC of the fifteen mRNAs (ALOX5, CARD6, CR1, FLOT1, HCK, HK3, ITGAM, LTB4R, MAPK14, NCF4, NLRC4, PGS1, PYGL, RAB31, SH3GLB1) and four lncRNAs (GSEC, NONHSAT160878.1, RARA-AS1 and XR_926068.1) was all above 0.88 in diagnosis of pediatric sepsis

**Figure 7.**
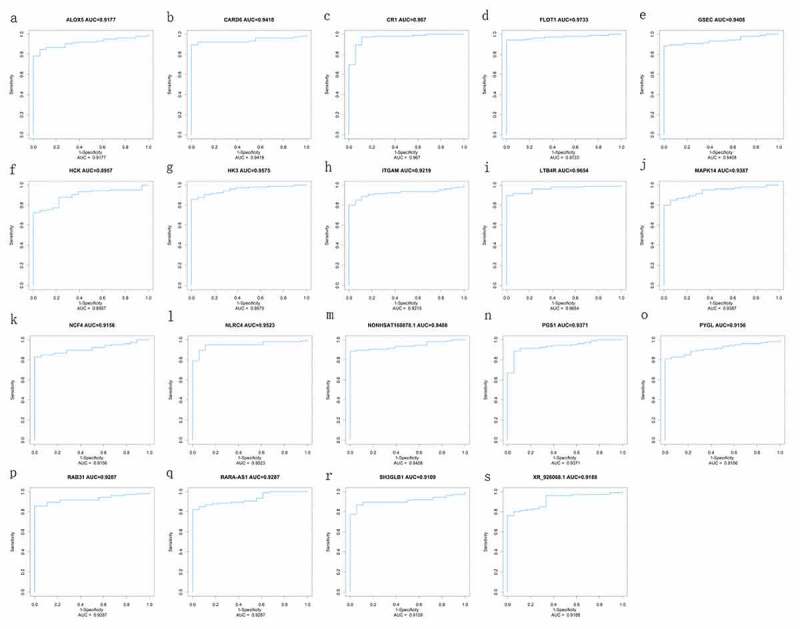
ROC curve of diagnosis of pediatric septic shock. the AUC of the fifteen mRNAs (ALOX5, CARD6, CR1, FLOT1, HCK, HK3, ITGAM, LTB4R, MAPK14, NCF4, NLRC4, PGS1, PYGL, RAB31, SH3GLB1) and four lncRNAs (GSEC, NONHSAT160878.1, RARA-AS1, and XR_926068.1) was all above 0.89 in diagnosis of pediatric septic shock

Using the identified 15 mRNAs (MAPK14, ITGAM, HK3, ALOX5, CR1, HCK, NCF4, PYGL, FLOT1, CARD6, NLRC4, SH3GLB1, PGS1, RAB31, LTB4R) and four lncRNAs (GSEC, NONHSAT160878.1, XR_926068.1, RARA-AS1), the ROC curves were constructed to estimate their specificity and sensitivity. The area under the curve (AUC) was above 0.88 between sepsis VS. control and septic shock VS. control groups, indicating that 15 mRNAs and four lncRNAs are likely to have good diagnostic value for pediatric sepsis (Figure 6 and Figure 7).

## Discussion

Early diagnosis of pediatric sepsis is a challenge but important for the effective treatment. And the mortality rate sharply increased when pediatric sepsis progressed to severe sepsis and septic shock. Many studies displayed the essential role of genetic and epigenetic alterations as it is related to sepsis diagnosis and treatment [15–17]. Therefore, molecular diagnosis can enhance the ability of rapid intervention and may decrease mortality rate.

In our study, 1941 mRNAs and 225 lncRNAs were identified based on GEO database. After WGCNA analysis, the turquoise module was significantly correlated with pediatric sepsis because of the maximum number of genes and significant *P* value. The mRNA‑lncRNA co‑expression network of the turquoise module provided an insight into the correlation between mRNA and lncRNA. And we also conducted mRNA‑lncRNA-Pathway co‑expression network and found 15 mRNAs and four lncRNAs were significantly up-regulated, which had positive function on inflammation and enriched in Complement and coagulation cascades, Phagosome, leukocyte trans-endothelial migration, metabolic pathways, Insulin signaling pathways, and other Inflammation or immune pathways. It indicated that pediatric sepsis was due to immune disorders caused by multiple genes, and also revealed the signal pathway that the four lncRNAs participate in. In addition, 15 mRNAs and four lncRNAs expected to be good diagnostic markers, because the AUC was all more than 0.88.

LncRNA participates in some important functions of cells and plays an important role in the pathogenesis of various diseases [18,19]. In the turquoise module, GSEC, NONHSAT160878.1 were core lncRNA linked to hub mRNAs (MAPK14, ITGAM, HK3, ALOX5, CR1, HCK, NCF4, PYGL, FLOT1, CARD6, RAB31). MAPK14 (Mitogen-activated protein kinase 14) could activate MAPK signaling pathway to aggravate acute lung injury (ALI) in septic shock mice [20]. The inhibition of MAPK14 also reduces the severity of ALI by inhibiting the activation of signaling pathway. ITGAM (integrin subunit alpha M) was essential for the activation and migration of inflammatory cells. It was found had high expression in adults and neonatal sepsis [21,22]. A previous study [23] had revealed that ITGAM mainly contributed to the progression of sepsis by promoting the nuclear, cytoplasmic translocation and activating release of HMGB1. ITGAM blocking antibodies or inhibitors could protect mice from the lethality associated with LPS and microbial sepsis [24]. HK3 (Hexokinase 3) phosphorylated glucose is used to produce glucose-6-phosphate and involved in the first step of glucose metabolism. The activity of HK3 increased rapidly after lipopolysaccharide (LPS) exposure, and the up-regulation of HK3 in sepsis was an important factor to interrupt energy production and cause AKI [25]. ALOX5 was an important enzyme for producing leukotrienes (LTs) to cause lung damage in sepsis [26]. ALOX5 products could induce pulmonary inflammation and its inhibition attenuated sepsis-induced lung injury [27]. CR1 (complement receptor 1) protected bacteria from leukocyte phagocytosis in human blood [28]. And CR1 monoclonal antibody could ameliorate sepsis-induced mortality during Staphylococcus aureus infection [29]. HCK belonged to Src family of tyrosine kinases. Its inhibitor reduced neutrophil migration and TNF-a secretion in mice and presented resistance to endotoxic shock. And it had an important role in transmitting LPS signaling in macrophages [30,31]. GSEC, NONHSAT160878.1 were co-expressed with these mRNAs. Thence, we speculated that GSEC, NONHSAT160878.1 might be involved in the progression of pediatric sepsis by regulating MAPK signaling pathway, TNF signaling pathway, Toll-like receptor signaling pathway, IL-17 signaling pathway, phagosome pathway, metabolic pathways, insulin signaling pathway, complement and coagulation cascades and chemokine signaling pathway to have potential function on pediatric sepsis.

XR_926068.1 was related to hub mRNAs (MAPK14, ALOX5, NCF4, FLOT1, NLRC4, SH3GLB1, PGS1). NLRC4 (NLR family CARD domain containing 4) was found to be up-regulated by MAPK pathway in pediatric sepsis, which also inhibited IL‑1β and IL‑18 production to contribute to the anti-inflammatory response [32]. SH3GLB1 was found as Bax binding protein at first, which is participated in autophagy and apoptosis [33]. PGS2 (phosphatidylglycerophosphate synthase 2) had a pro-inflammatory effect [34]. In this study, XR_926068.1 was co-expressed with these mRNAs. So it might be involved in the progression of pediatric sepsis by regulating NOD-like receptor signaling pathway, autophagy and metabolic pathways to have a regulation effect on pediatric sepsis.

RARA-AS1 was connected with hub mRNAs (ITGAM, HK3, ALOX5, NCF4, FLOT1, LTB4R). NCF4 promoted intracellular ROS production and played a role in immune and inflammatory diseases [35,36]. FLOT1 had been reported to increase the risk of fungal infection in stem cell transplant recipients [37]. LTB4R (Cytokines and leukotriene B4 receptor) was viewed as pro-inﬂammatory mediators, and its down-regulated expression could attenuate microcirculation impairment during sepsis [38]. LTB4/LTB4R pathway was also enriched in the pathogenesis of the sepsis [39]. RARA-AS1 was co-expressed with these mRNAs. Then, we speculated that RARA-AS1 might be involved in the progression of pediatric sepsis by regulating phagosome, leukocyte transendothelial migration, and insulin signaling pathways.

## Conclusion

In conclusion, our study highlights the important roles of turquoise module in the progression of pediatric sepsis. This article comprehensively expounds the mRNA and lncRNA differences between pediatric sepsis and normal group. Fifteen mRNAs (MAPK14, ITGAM, HK3, ALOX5, CR1, HCK, NCF4, PYGL, FLOT1, CARD6, NLRC4, SH3GLB1, PGS1, RAB31, LTB4R) and four lncRNAs (GSEC, NONHSAT160878.1, XR_926068.1 and RARA-AS1) were found play important roles on pediatric sepsis by WGCNA analysis. And they all have good diagnostic efficiency in pediatric sepsis and septic shock. It provides more directions to study the useful diagnostic markers and molecular mechanism of pediatric sepsis. Several limitations still existed in this study. Large numbers of pediatric sepsis samples are needed for further researches and more experiments are required to understand the biological function of key lncRNAs.

## Supplementary Material

Supplemental Material

## Data Availability

The datasets during the current study are available in the GEO: https://www.ncbi.nlm.nih.gov/geo/query/acc.cgi?acc=GSE13904. Links to repositories for the annotation, visualization and integrated discovery are available at DAVID: http://david.abcc.ncifcrf.gov/.

## Highlights

1. Turquoise module is significantly correlated with pediatric sepsis.
2. The module co-expression network is enriched with inflammatory signal pathways.
3. Fifteen mRNAs and four lncRNAs are identified to be potential diagnostic markers for pediatric sepsis.
