# Supplementary material for: Analysis of mRNA‑lncRNA and mRNA‑lncRNA-pathway co‑expression networks based on WGCNA in developing pediatric sepsis
Source: Bioengineered. 2021 May 5;12(1):1457–70. doi: 10.1080/21655979.2021.1908029 (PMC8806204; doi:10.1080/21655979.2021.1908029)
Supplement: Supplemental Material [file KBIE_A_1908029_SM3427.zip › Document.rtf]

Supplementary information

4 Supplemental Figure 1. Cluster graph of differential mRNA expression levels change in (a)
5 control vs. pediatric sepsis day1, (b) control vs. pediatric sepsis day3, (c) control vs. pediatric
6 septic shock day1, (d) control vs. pediatric septic shock day3, (e) control vs. pediatric SIRS
7 day1, (f) control vs. pediatric SIRS day3 and (g) control vs. pediatric SIRS resolved day3
8 groups. Each column of Cluster graph represents a sample and each row represents a
9 differential gene. Red represents a relatively high expression, and green represents a relatively
10 low expression.

11 Supplemental Figure 2. Cluster graph of differential lncRNA expression levels change in (a)
12 control vs. pediatric sepsis day1, (b) control vs. pediatric sepsis day3, (c) control vs. pediatric
13 septic shock day1, (d) control vs. pediatric septic shock day3, (e) control vs. pediatric
14 SIRSday1, (f) control vs. pediatric SIRS day3 and (g) control vs. pediatric SIRS resolved
15 day3 groups. Each column of Cluster graph represents a sample, and each row represents a
16 differential lncRNA. Red represents a relatively high expression, and green represents a
17 relatively low expression.
