# Supplementary figures and images for: Analysis of mRNA‑lncRNA and mRNA‑lncRNA-pathway co‑expression networks based on WGCNA in developing pediatric sepsis
Source: Bioengineered. 2021 May 5;12(1):1457–70. doi: 10.1080/21655979.2021.1908029 (PMC8806204; doi:10.1080/21655979.2021.1908029)

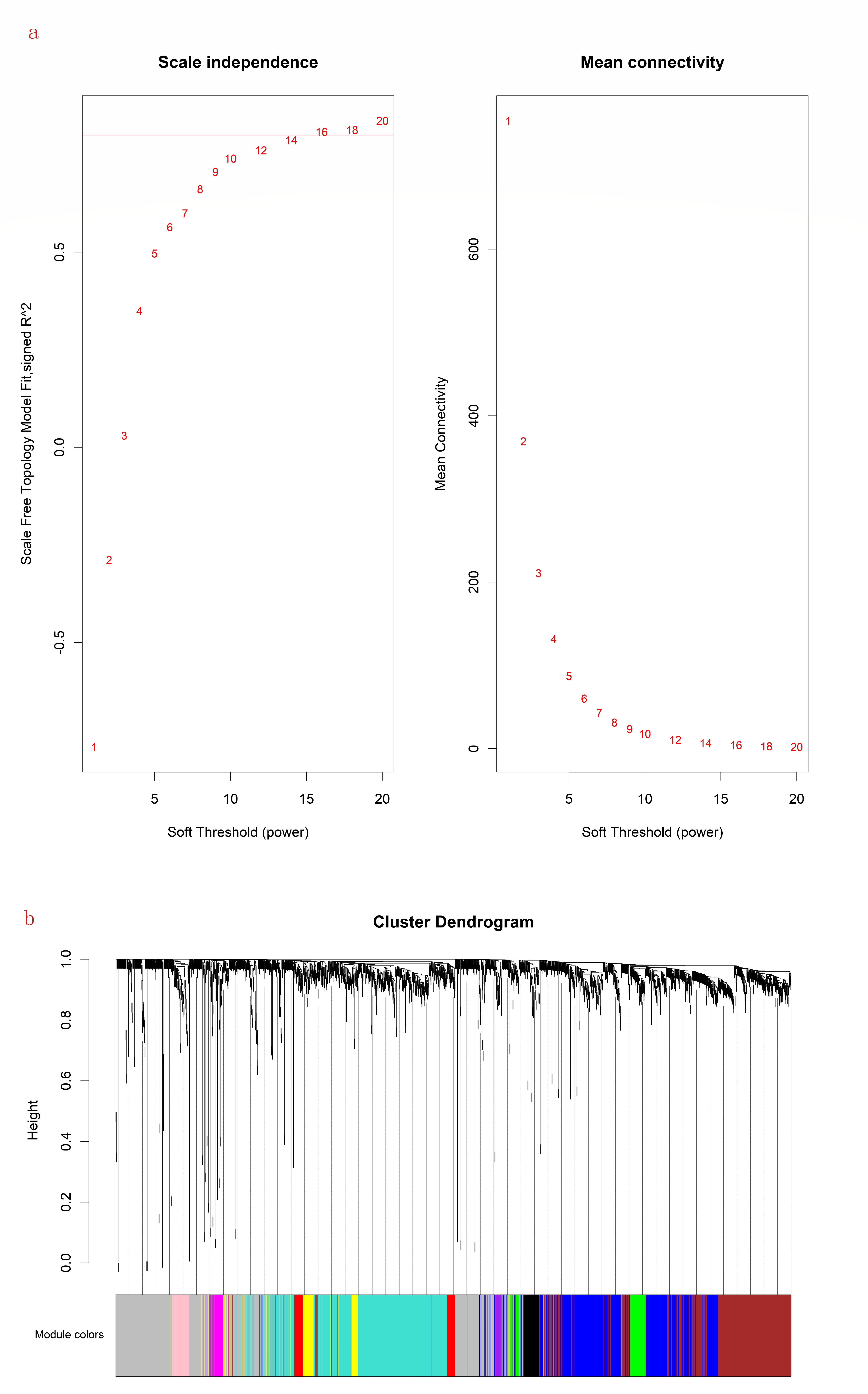

Supplement: Supplemental Material [file KBIE_A_1908029_SM3427.zip › supplemental Fig 1.jpg]

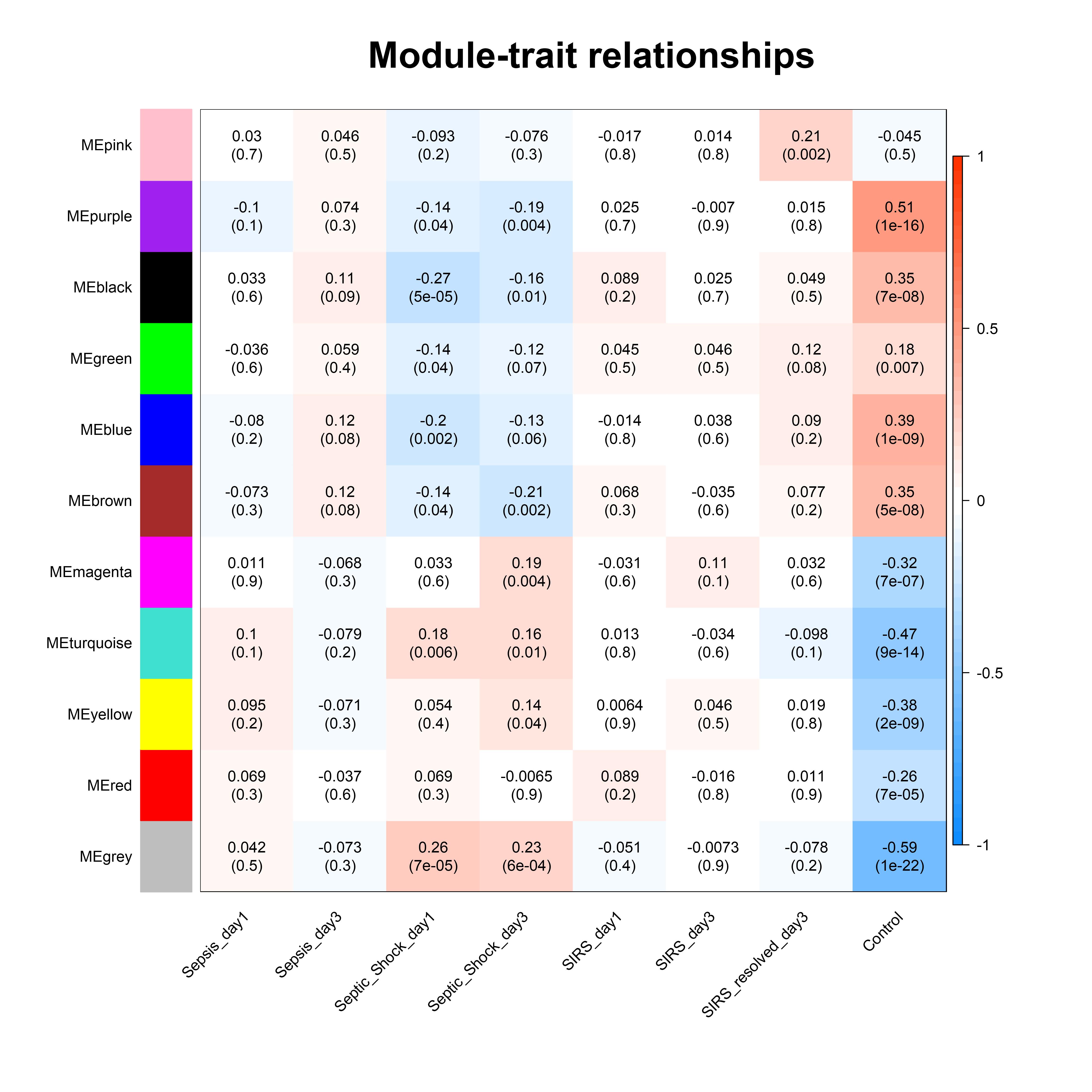

Supplement: Supplemental Material [file KBIE_A_1908029_SM3427.zip › supplemental Fig 2.jpg]
